# Mean 5-Year Clinical and Radiographic Outcomes of Cementless Total Hip Arthroplasty in Patients under the Age of 30

**DOI:** 10.1155/2013/649506

**Published:** 2013-06-24

**Authors:** Jeremy M. Gililland, Lucas A. Anderson, Jill Erickson, Christopher E. Pelt, Christopher L. Peters

**Affiliations:** Department of Orthopaedic Surgery, University of Utah, 590 Wakara Way, University Orthopaedics Center, Salt Lake City, UT 84108, USA

## Abstract

We performed a retrospective review of 40 consecutive modern cementless THAs with 65-month mean followup in 34 patients under the age of 30 primarily for diagnoses other than inflammatory arthritis. We found acceptable functional improvement and radiographic outcomes at mean 5-year followup. We found a high transfusion rate, dislocation rate (10%), and midterm overall aseptic revision rate (17%). Twenty-eight (67.5%) of hips in this series were metal on metal, with a large percentage of aseptic revisions related to metallosis (57%). When revisions due to metallosis were excluded, the aseptic revision rate was 7.5%. The high prevalence of prior pediatric hip surgery in these patients (50%) may predispose to increased technical difficulty resulting in increased complications and higher revision rates. Although our revision rate was high in these young patients, it is favorable compared to older techniques and consistent with the limited data available with modern cementless techniques in patients of similar age. Cementless THA with modern designs remains a viable option for the treatment of arthritis in the young patient.

## 1. Introduction

Total hip arthroplasty (THA) is a highly successful surgical intervention as it restores function, alleviates pain, and greatly improves quality of life. Despite the overwhelming success of this surgical intervention, surgeons may be averse to recommend THA in the young patient due to anticipated high activity level resulting in repetitive loading, excessive demand placed on the hip, and the limited implant survivorship. Moreover, surgical treatment of this patient population is challenging due to the often aberrant proximal femoral geometry, previous surgery with retained hardware, leg length discrepancy, and relative acetabular dysplasia or retroversion.

To date, there remains a paucity of studies assessing the efficacy of THA in the young patient. Moreover, of the available studies, many discuss the outcomes of THA in patients with inflammatory arthritis [1–3] leaving few studies describing the outcome of THA in noninflammatory hip degeneration [4–11]. From an implant standpoint, the primary concern is the increased risk of failure and high likelihood for revision surgery in the patient's lifetime, making the use of cementless implants appealing as the likelihood for aseptic loosening is decreased and stable long-term fixation is expected [10–12]. Additionally, if the implant becomes loose, the revision procedure is technically more facile as the surgeon does not need to remove cement.

A review of the literature shows that in an older population, the use of proximally coated, tapered cementless stems in combination with modern articulations has excellent survivorship approaching 95% at 20 years [5]. As a regional referral center with an interest in young adult hip preservation in addition to arthroplasty, we frequently encounter young patients with end stage hip osteoarthritis and consequently are often left with the clinical dilemma of the most appropriate treatment in this age group. Herein, we present our radiographic and clinical results following total hip arthroplasty in a series of young patients under the age of 30 with advanced coxarthrosis primarily secondary to noninflammatory processes using modern cementless implants and bearing couples.

## 2. Materials and Methods

After IRB approval, we performed a retrospective review of 40 consecutive cementless total hip arthroplasties performed by a single surgeon (CLP) from 1996–2008 in 34 patients under the age of 30 with mean followup of 65 months (range 24–151). The components utilized in all cases were a cementless acetabular component and a proximally porous coated cementless femoral component. The femoral components included a tapered wedge design (Taperloc, Biomet, Warsaw, IN), or a modular S-ROM (Depuy, Warsaw, IN). Acetabular components consisted of monoblock cobalt chrome (CoCr) cups (M2a or Magnum, Biomet, Warsaw IN or ASR, Depuy, Warsaw, IN), modular titanium cups with a CoCr insert (Pinnacle, Depuy, Warsaw, IN), or modular titanium cups with a conventional, highly cross-linked or vitamin-E enhanced cross-linked polyethylene liner (Ranawat-Burnstein Ringloc with Arcom, ArcomXL or E1 polyethylene (Biomet, Warsaw, IN) (Table 1). Our primary outcome measures included the Harris hip score [15], clinical complications, revisions, and radiographic analysis focusing both on femoral and acetabular radiolucencies.

All surgeries were performed in a clean-air operating room, and the operating team wore body-exhaust suits. All patients received prophylactic intravenous antibiotics 30 to 60 minutes prior to incision and which were continued for 24 hours after surgery. A standard miniposterolateral approach was used in the majority of cases, though direct lateral or anterolateral approaches were also utilized. All acetabular components were inserted using a standard 1-2 mm press-fit technique. Adjunct acetabular screws were used as needed to achieve initial mechanical stability of the acetabular component. Patients received pharmacologic venous thromboembolic prophylaxis for at least 4 weeks after surgery with low molecular weight heparin or warfarin. Patients were made weight bearing as tolerated in the immediate postoperative period unless contraindicated based upon an intraoperative complication.

Patients were evaluated prior to the index surgery and scheduled postoperatively at six weeks, six months, one year, and biannually thereafter with clinical exam, HHS, and radiographs. The patient records were reviewed to obtain patient demographic data, preoperative diagnosis, operative details, and arthroplasty component information. Preoperative and postoperative Harris hip scores (HHSs) from the latest followup visit are reported [15]. Serial radiographic evaluation included anteroposterior pelvis and groin lateral films of the operative side. Two reviewers (LAA, JMG) evaluated the most recently obtained radiographs of the hip for component migration or subsidence and radiolucencies in acetabular zones of De Lee and Charnley [16] and femoral zones of Gruen [17]. All postoperative complications and any revisions were documented prospectively in our total joint database.

## 3. Results

The mean age of this group of patients was 22 years (range 15–29) with a mean BMI of 27 (range 19–43). 50% of these patients had prior ipsilateral hip surgery, and 47.5% were Charnley class B or C. The majority of these patients had a preoperative diagnosis of pediatric hip disease (52.5%) or avascular necrosis (30%), and only 3 patients (7.5%) had a diagnosis of inflammatory arthritis (Table 1).

In terms of perioperative factors, we noted a relatively high estimated blood loss (EBL) (mean 456 mL, range 200–1200) and rate of transfusions (15%). The majority of these cases were done through a posterior approach (75%). In regards to component utilization, and likely related to the rate of prior hip surgery, we noted a large amount of modular femoral stems in the young group (42.5%). The majority of articulations in these patients were metal on metal (MOM) (67.5%), with 89% of these MOM bearings utilizing large heads (head size 36 mm or greater) and 79% utilizing monoblock acetabular components (Table 1).

The overall perioperative complications rate was 15%. Dislocation accounted for 67% of these complications, with 10% of these younger patients having had at least one dislocation. Other complications included infection in one patient and pulmonary embolus in one patient (Table 2).

Clinical outcomes were measured using the harris hip score (HHS). We found a mean 33.4 point (95% CI: 28.0–37.6) improvement in the Harris Hip Score following THA, with mean preoperative HHS of 62.7 (95% CI: 57.3–68.0) and a mean postoperative score of 94.7 (95% CI: 92.2–97.1). The most recent followup AP and lateral radiographs of the hip were evaluated for femoral and acetabular radiolucencies. In terms of radiographic outcomes, we identified Zone 1 femoral radiolucencies in 4 femurs (10%) and a Zone 2 radiolucency in 1 femur (2.5%). We did not identify any other femoral radiolucencies. On the acetabular side, we found Zone 1 radiolucencies in 2 acetabuli (5%), a Zone 2 radiolucency in 1 acetabulum (2.5%), and a Zone 3 radiolucency in 1 acetabulum (2.5%) (Table 2). No radiolucencies were progressive, and one implant was deemed radiographically loose.

Our aseptic revision rate was 17.5% with a mean time to revision of 74 months (range 22–124 months) (Table 2). Due to the large number of metal on metal articulations in this series, we divided our aseptic revisions into revisions for metallosis and revisions for reasons other than metallosis. Metallosis accounted for the majority of our aseptic revisions (57%), and when excluding these cases, we found three aseptic revisions for a rate of 7.5%, with one patient revised for instability, one for periprosthetic femur fracture, and one for aseptic loosening of both the femoral and acetabular components (Table 3).

## 4. Discussion

Surgeons are frequently presented with the clinical dilemma as to the most appropriate treatment option for young patients with advanced coxarthrosis. Much of the existing literature available on total hip arthroplasty in the young patient describes the results when performed for inflammatory arthropathies. Additionally, the majority of early studies involved the use of cemented fixation in these young patients. Many studies evaluating outcomes of cemented total hip arthroplasty in patients less than 30 years of age have shown poor results with high revision rates [18, 19]. In a 1984 study, with an overall aseptic revision rate of 33% at 8-year followup of cemented THAs in patients less than 20 years of age with diagnoses of polyarticular inflammatory arthritis, Roach insightfully noted that “perhaps in the future, non-cemented prostheses may better serve this difficult group of patients” [19]. Subsequently, in contrast to cemented fixation, several studies were published showing much improved clinical outcomes and aseptic revision rates with cementless THA in these younger patients with polyarticular disease [1–3]. However, in his study of THAs in patients less than 30 years of age, Chandler noted higher revision rates in patients with unilateral arthroplasties and higher activity levels as compared to those with polyarticular inflammatory disease [18]. The improved outcomes in the patients with juvenile inflammatory conditions are thought to be secondary to decreased activity levels due to their polyarticular disease, and this disparity has made it difficult to translate these published results to young patients without inflammatory arthritis. With the present study, we add our results to the limited body of the literature on the use of contemporary cementless total hip arthroplasty with mid- to long-term followup in young patients (<30 years) with primarily noninflammatory coxarthrosis (Table 4).

An examination of the literature shows a common finding of higher rates of revision for aseptic loosening in this younger THA population consistent with the trend found in this study (Table 4). Nizard et al., Dudkiewicz et al., and Wangen et al. all found very high rates of revision for aseptic loosening in the their cohorts (range 15 to 45%) [6, 9, 11], while Kamath et al., Costa et al., Clohisy et al., Restrepo et al., and our current study found rates from 0–8% [4, 5, 8, 14]. When evaluating the available literature with a majority of noncemented THAs in young patients with primarily non-inflammatory arthritis, we can see that the aseptic revision rates are improved compared to the traditionally high revision rates in this young population performed with other techniques [18, 19]. However, there does seem to be a trend toward drastically rising revision rates as followup increases (Table 4). In most of these studies, a higher rate of loosening was found on the acetabular side compared to the femoral component (3 : 1), while our revision rates were similar on both the femoral and acetabular sides (Table 4). While our aseptic femoral revision rate does seem high at 5%, one of these revisions was in the setting of a periprosthetic fracture and only one of these femoral revisions was for aseptic loosening of the stem. If this is taken into account, our aseptic femoral revision rate would have been 2.5% at 65-month followup which is similar to recent metaanalysis data finding aseptic revision rates of cementless femoral components in younger patients to be 1.3% at mean 8.4-year followup [20].

This study is one of the only available studies looking at a group of young patients having had THA with a majority of modern large head MOM bearings (70%). A recent study by Girard et al. found low revision rates (4.3% at 9 years) compared to our MOM THA patients (17.5% overall and 10% for metallosis alone at 5.4 years) [7]. However, they were using a metal-polyethylene sandwich acetabular bearing surface with smaller heads (89% 28 mm heads), which has not shown the same revision rates as other large head monoblock metal-on-metal bearings. In other published series, these metal-polyethylene sandwich bearings have had similar revision rates to standard metal-on-polyethylene bearings [21–24]. Therefore, we feel that our metallosis revision rate of 10% at 5.4 years may be a more accurate reflection of actual revision rates in young patients with large head monoblock metal-on-metal articulations.

In order to further put our results into perspective, we performed a comparison of this young group of patients to an internal control group of 50 randomly selected cementless total hip arthroplasties performed in 49 patients over the age of 50 during this same time period matched for the duration of followup and the distribution of the bearing utilized. In a post-hoc power analysis, we found that we were grossly underpowered for most of the comparisons between these groups, and we therefore do not formally present this data as a comparative retrospective study. However, despite not meeting statistical significance, we did find several interesting findings that we feel are clinically significant and an accurate description of our experience and thus should be highlighted. Among the demographic details, as might be expected, our young patients had a smaller mean BMI of 27 compared to 32 in the older patients (*P* = 0.033). We also noted a trend toward more Charnley A hips in the older group (68% versus 52.5%), which is also logical as more of the younger group had diagnoses of sequelae of pediatric diseases, which were often bilateral. Further, and again not surprising is the fact that more of the young patients had a diagnosis of avascular necrosis or osteoarthritis secondary to prior pediatric hip diseases, while the majority of our older patients had a diagnosis of idiopathic osteoarthritis. Both groups had very similar preoperative mean Harris hips scores. In terms of components utilized, we found more modular femoral stems in the young group (43% versus 4%), likely as a result of the altered or small anatomy encountered in these patients with the sequelae of pediatric hip diseases. Among our perioperative data, we noted a trend toward more blood loss (456 versus 403 mL) and postoperative transfusions (15% versus 6%) in the younger group. We are unsure of the reason for the higher transfusion rate seen in the younger group as younger patients are often more tolerant of lower hemoglobin levels. Our transfusion trigger was not specifically different between the groups. We believe that this higher transfusion rate may indicate that our trend toward more blood loss in the younger group may have been an underestimate of the increased blood loss actually seen, especially since intraoperative estimations of blood loss are usually quite subjective. This increased blood loss may be a reflection of the fact that 50% of our young hips had prior surgery making the THA more difficult requiring more extensile approaches and more blood loss. We also found a trend toward more dislocations (10% versus 2%) and thus overall complications (15% versus 6%) among the younger group. When looking at complications other than dislocation, the rate of complications was similar between the groups (5% versus 4%). Among our outcomes data, we found a trend in the younger group of higher overall aseptic revision rates (17.5% versus 8%) and aseptic revisions for reasons other than metallosis (7.5% versus 4%). When we excluded failures for metallosis, our midterm aseptic revision rates in both groups were similar to the existing literature and we feel that this strengthens this comparison despite statistical insignificance [13, 25]. In terms of both radiographic and clinical outcomes, the two groups were notably similar.

There are several limitations to this study. The retrospective nature yields itself to several biases, including selection and recall bias. The followup on this study is a limitation in that our mean followup is only 5.4 years, which limits conclusions regarding long-term clinical outcomes, complications, and need for further interventions. Additionally, our young THA cohort had limited numbers, making an attempt to compare this to a control group grossly underpowered. However, despite our limited power, this comparison does provide some unique information as none of the other existing literature provides any comparison to an internal control group of older patients, and we have therefore included our findings above.

In conclusion, contemporary cementless THA in patients under the age of 30 for diagnoses other than inflammatory arthritis is associated with acceptable functional improvement and radiographic outcomes at midterm followup. There does appear to be a trend toward a higher transfusion rate, higher dislocation rate, and higher midterm overall aseptic revision rate in this young group of patients. The high prevalence of prior pediatric hip surgery in the young THA group may predispose to increased technical difficulty resulting in increased complications and higher revision rates in these younger patients. Although our revision rate was high in the younger patients, it is favorable compared to older techniques and consistent with the limited data available with modern cementless techniques in patients of similar age. Long-term followup of this important group of patients will be imperative to evaluate longevity and the sequelae of implant wear in these patients who are likely to outlive their prosthetic bearings.

## Figures and Tables

**Table 1:** Patient demographics, perioperative data, and component data.

Demographics	
Age at surgery [mean (range)]	22 (15–29)
Gender	24 F (60%)
	16 M (40%)
BMI [mean (range)]	27 (19–43)
Side	19 L (47.5%)
	21 R (52.5%)
Prior-hip surgery	20 (50%)
Charnley class A	21 (52.5%)
Charnley class B	10 (25%)
Charnley class C	9 (22.5%)

Diagnosis	

OA secondary to pediatric hip diseases	21 (52.5%)
Avascular necrosis (AVN)	12 (30%)
Inflammatory arthritis	3 (7.5%)
Failed hip fusion	2 (5%)
Septic arthritis	1 (2.5%)
Posttraumatic arthritis	1 (2.5%)

Perioperative data	

Estimated blood loss [mean (95% CI)]	456 (374–537)
Postoperative transfusions	6 (15%)
Intraoperative fractures	2 (5%)
Posterior approach	30 (75%)
Anterolateral approach	8 (20%)
Direct lateral approach	2 (5%)

Femoral component	

Modular cementless stem	17 (42.5%)
Nonmodular cementless stem	23 (57.5%)

Acetabular component	

Monoblock CoCr Cup	21 (52.5%)
Titanium cup with coCr insert	6 (15%)
Titanium cup with polyethylene insert	13 (32.5%)

Articulation	

Metal-on-metal	27 (67.5%)
Metal-on-conventional polyethylene	11 (27.5%)
Metal-on-highly cross-linked polyethylene	1 (2.5%)
Metal-on-vitamin E cross-linked polyethylene	1 (2.5%)

Femoral head size	

22 mm to 28 mm	14 (35%)
32 mm to 38 mm	12 (30%)
40 mm to 56 mm	14 (35%)

**Table 2:** Complications, revision rates, and radiographic and Clinical outcomes.

Complications	*n* (%)
Infection	1 (2.5%)
Pulmonary embolism	1 (2.5%)
Dislocation	4 (10%)
Total complications	6 (15%)

Revisions	*n* (%)

All aseptic revisions	7 (17.5%)
Time to revision [mean (95% CI)]	74 (38–110)

Radiographic outcomes	*n* (%)

Femoral radiolucencies (gruen zones)	
Zone 1	4 (10)
Zone 2	1 (2.5)
Zone 3	0
Zone 4	0
Zone 5	0
Zone 6	0
Acetabular radiolucencies (De Lee and Charnley zones)	
Zone 1	2 (5)
Zone 2	1 (2.5)
Zone 3	1 (2.5)

Clinical outcomes	mean (95% CI)

Preop Harris hip score [mean (95% CI)]	62.7 (57.3–68.0)
Postop Harris hip score [mean (95% CI)]	94.7 (92.2–97.1)
Change in Harris hip score [mean (95% CI)]	33.4 (28.0–37.6)

**Table 3:** Revision details (7 aseptic revisions—17.5% aseptic revision rate at mean 65-month mean followup).

Diagnosis	Charnley class	Femoral head type	Femoral head size	Femoral component	Acetabular component	Articulation	Reason for revision	Revision details	Time to revision (months)
AVN	B	CoCr	22	Biomet Integral	Biomet RB	MOP	Recurrent dislocations-poly wear.	Exchanged liner and head.	123.7

JRA	C	CoCr	22	DePuy SROM	Biomet RB	MOP	Periprosthetic femur fracture.	Stem revised.	92.5

OA 2/2 Perthes	A	CoCr	45	DePuy SROM	DePuy ASR	MOM-ASR	Aseptic loosening. No metallosis.	Stem loose, fibrous fixation cup. Complete revision with MOP.	26.7

Failed hip fusion (Fused for AVN)	A	CoCr	28	Biomet MH Calcar	Biomet RB	MOM (sandwich)	Recurrent dislocation. Metallosis found.	Cup and stem stable. Revised to MOP.	101.8

AVN	A	CoCr	38	Biomet Intergral	Biomet M2A	MOM-M2A	Metallosis-mild metal debris/excessive scar.	No loosening. Revised to MOP	92.1

OA 2/2 CDH	A	CoCr	41	DePuy SROM	DePuy ASR	MOM-ASR	Metallosis.	No loosening. Revised to MOP.	60.8

OA 2/2 CDH	A	CoCr	46	DePuy SROM	DePuy ASR	MOM-ASR	Metallosis.	No loosening. Revised to MOP	21.8

AVN: avascular necrosis, JRA: juvenile rheumatoid arthritis, OA: osteoarthritis, CDH: congenital dysplasia of the hip, CoCr: cobalt chromium, MOM: metal on metal, MOSP: metal on conventional polyethylene.

**Table 4:** Studies of patients <30 years old with majority noninflammatory arthritis treated with cementless total hip arthroplasty (sorted by mean followup).

Study	Kamath et al. [8]	Costa et al. [5]	Clohisy et al. [4, 13]	Current Study	Restrepo et al. [10, 14]	Nizard et al. [9]	Dudkiewicz et al. [6]	Girard et al. [7]	Wangen et al. [11]
Year pub	2012	2012	2010	2013	2008	2008	2003	2010	2008

Mean age (range)	18 (13–20)	20 (13–30)	20 (12–25)	22 (15–29)	17.64 (13.5–20)	23.4 (13–30)	23.2 (14–29)	25 (15–30)	25 (15–30)

Patients (hips)	17 (20)	40 (53)	88 (102)	34 (40)	25 (35)	94 (108)	56 (69)	34 (47)	44 (49)

Mean F/U (years)	4.1	4.6	5	5.4	6.6	6.9	7.4	9	13

Prior operations	80%*	0%	Unknown	50%	20%	18%	High	27.70%	Unknown

% Inflammatory arthritis	0%	0%	12%	7%	26%	13%	29%	4%	0%

Surgical approach	100% post	100% AL	Post and AL	75% post 20% AL 5% DL	100% AL	90% Post 6% DL 4% AL	Unknown	100% post	Post and DL

Cementless stems	95%	100%	95%	100%	100%	53%	91.30%	94%	100%

Cementless cups	100%	100%	100%	100%	100%	92%	91.30%	100%	100%

Articulation	70% COC 30% MOXLP	100% MOP	45% MOXLP 29% MOP 14% COC 7% COXLP 5% MOM	67.5% MOM 27.5% MOP 5% MOXLP 5% MOEP	63% COXLP 31% MOP 6% COC	100% COC	100% MOP	100% MOM (Metasul)	100% MOP

Infections	0%	1.9%	1%	2.5%	0%	0%	4.3%	4.3%	Unknown

Dislocations	0%	0%	3.9%	10%	0%	1.9%	7.2%	2.1%	6.1%

Femoral aseptic Revision rate	0%	0%	0%	5%**	0%	4.6%	4.3%	0%	0%

Acetabular aseptic revision rate	0%	1.9%	2.9%	2.5%**	0%	13.9%	15.9%	4.3%	49%

Overall aseptic revision rate	5%	3.8%	7%	7.5%**	0%	15.7%	15.9%	4.3%	49%

*Majority of prior operations were core decompression procedures for avascular necrosis.

**Failure rates for reasons other than metallosis are listed for the current study.

Post: posterior approach, AL: anterolateral approach, DL: direct lateral approach.

COC: ceramic on ceramic, MOXLP: metal on highly cross-linked polyethylene, MOP: metal on polyethylene, MOEP: metal on E-polyethylene, COXLP: ceramic on highly cross-linked polyethylene, MOM: metal on metal.
